# Supplementary material for: Potential Methods of Targeting Cellular Aging Hallmarks to Reverse Osteoarthritic Phenotype of Chondrocytes
Source: Biology (Basel). 2022 Jun 30;11(7):996. doi: 10.3390/biology11070996 (PMC9312132; doi:10.3390/biology11070996)
Supplement: Supplementary file 1 [file biology-11-00996-s001.zip › biology-1688636-supplementary.pdf]

Table S1. Potential strategies targeting different hallmarks of cellular aging. SASPs: senescence associated secretory phenotypes; ROS: reactive oxygen species; MMR: mismatch repair system; DAMPs: damage associated molecular patterns; ER: endoplasmic reticulum; NAD: nicotinamide adenine dinucleotide; SOD2: superoxide dismutase 2; Nrf2: nuclear factor-erythroid 2-related factor; MSC: mesenchymal stem cell; iPSC: induced pluripotent stem cell; PRR: pattern recognition receptor; CR: calorie restriction; PI3K: phosphatidylinositol 3 kinase; HMG-CoA: 3-hydroxy-3-methyl glutaryl coenzyme A reductase; eIF: eukaryotic translation initiation factor; HSF: heat shock factor; SQST: sequestosome; GH: growth factor; IGF: insulin-like growth factor.

Target	Strategy	Typical options	Ref.
Telomere elongation	Transfer telomerase gene	Transfecting telomerase gene	167
	Re-express telomerase gene	Cycloastragenol	168-171
	Re-active residential telomerase	AGS-499	173
	Modulate telomerase location	Modulating the intracellular location	166
Anti-cellular senescence	Eliminate senescent cells	ABT-263 (navitoclax), ABT737, dasatinib, quercetin, 2-deoxyglucose, FOXO4 D-retro inverso isoform.	235-243
	Supply senomorphics	Fisetin, rapamycin, ruxolitinib, loperamide, niguldipine, apigenin, and kaempferol.	91,245
	Suppress SASPs production	Heterochronic parabiosis; MSC- and iPSC-based therapies.	247,249
Epigenetic modification	Apply caloric restriction	Restricting calories in dietary.	184-186
	Deacetylate histone by Sirtins	Increasing expression levels of Sirtuins, especially SIR2 and its homologues.	181,182
	Transfer ncRNAs	Transfecting miR-140, miRNA-222; Supplying SAHA (vorinostat) and LBH589 (panobinostat).	195,197,198
Genomic stability maintenance	Eliminate ROS	Supplementing antioxidative agents such as Wogonin, ascorbic acid and NAD^+ precursors.	164
	Ameliorate DNA MMR system	Elevating zinc finger protein 16 and the inhibitor of nuclear factor kappa-B kinase.	123,163
	Restore antioxidative capacity	Activating antioxidative genes such as SOD2, and Nrf2.	124,153,154
Altered intercellular communication	Alleviate Inflammation	Eliminating SASPs and inflammasomes.	250,251
	Eliminate DAMPs	Depressing the expression or inhibiting the transduction of DAMPs or PRRs.	256,257
	Use bioengineered exosomes	Applying natural or bioengineered exosomes.	255
Mitochondrial function regulation	Recover glycolysis state	-	-
	Supply dietary antioxidants	CoQ10	199
	Keep mtDNA copy number	A-769662	200,207

	Remove damaged mitochondria	Trehalose	203-205
	Promote mitochondrial biogenesis	CGS21680	202,208
	Maintain mitochondrial proteostasis	T5224	210
	Decrease ROS production	Spermidine	201
	Restore membrane potential	Dimethyloxalylglycine	209
Metabolism interventions	Restrict calorie intake	Applying CR, Mediterranean diet, intermittent fasting, and prolonged fasting.	219,221-223
	Supply dietary nutraceuticals	Supplying nutraceuticals such as anthocyanidins, resveratrol, quercetin, flavonols and curcumin.	224,225,283
	Suppress anabolic activities	Supplying rapamycin, metformin, GH/IGF1 axis, and PI3K inhibitors; Intaking strawberries and blueberries.	42,230,231
	Intake pharmacological and natural antioxidants	N-acetylcysteine, α-tocopherol, HMG-CoA reductase inhibitors Ginkgo biloba.	174,175
Proteostasis restoration	Promote autophagy and mitophagy	Deleting eIF-1, eIF4E, eIF4G, eIF4A, eIF2B, eIF5A; Promoting EFK-1 and HSF-1.	213,214
	Improve proteasome and lysosomal function	Upregulating p62/ SQST-1.	217
	Relieve ER stress	Suppressing IGF-1 and supplying rapamycin.	211,212
